# Astaxanthin protects against early burn-wound progression in rats by attenuating oxidative stress-induced inflammation and mitochondria-related apoptosis

**DOI:** 10.1038/srep41440

**Published:** 2017-01-27

**Authors:** Quan Fang, Songxue Guo, Hanlei Zhou, Rui Han, Pan Wu, Chunmao Han

**Affiliations:** 1 Department of Burns, Second Affiliated Hospital, Zhejiang University, College of Medicine, 88 Jiefang Road, Hangzhou 310009, Zhejiang, China; 2 Department of Plastic Surgery, Binjiang Branch, Second Affiliated Hospital, Zhejiang University, College of Medicine, 1511 Jianghong Road, Hangzhou 310000, Zhejiang, China; 3 Department of Dermatology, Sir Run Run Shaw Hospital, School of Medicine, Zhejiang University, 3 East Qingchun Road, Hangzhou 310020, Zhejiang, China

## Abstract

Burn-wound progression can occur in the initial or peri-burn area after a deep burn injury. The stasis zone has a higher risk of deterioration mediated by multiple factors but is also considered salvageable. Astaxanthin (ATX), which is extracted from some marine organisms, is a natural compound with a strong antioxidant effect that has been reported to attenuate organ injuries caused by traumatic injuries. Hence, we investigated the potential effects of ATX on preventing early burn-wound progression. A classic “comb” burn rat model was established in this study for histological and biological assessments, which revealed that ATX, particularly higher doses, alleviated histological deterioration in the stasis zone. Additionally, we observed dose-dependent improvements in oxidative stress and the release of inflammatory mediators after ATX treatment. Furthermore, ATX dose-dependently attenuated burn-induced apoptosis in the wound areas, and this effect was accompanied by increases in Akt and Bad phosphorylation and a downregulation of cytochrome C and caspase expression. In addition, the administration of Ly 294002 further verified the effect of ATX. In summary, we demonstrated that ATX protected against early burn-wound progression in a rat deep-burn model. This protection might be mediated by the attenuation of oxidative stress-induced inflammation and mitochondria-related apoptosis.

Deep burn wounds are not usually stable and undergo a dynamic progress that results in the deepening and expansion of the initial burn area, a process defined as burn-wound progression^1^. This progressive damage can induce the conversion of the burn wound from a superficial partial-thickness burn to a deep partial-thickness or full-thickness burn and the development of initially unburned skin into part of the burn wound. DM Jackson first introduced the theory of the presence of three concentric zones in burn wound tissue: the zones of coagulation, stasis and hyperemia, from the central to the outer space^2^. The zone of stasis is considered salvageable, even though it faces a high risk of hypoperfusion, which can cause progressive necrosis during the early stages following burn injuries^3^. Moreover, the zone of coagulation undergoes irreversible necrosis caused by direct thermal injury, and the zone of hyperemia is usually recoverable^3^. Therefore, the zone of stasis is considered a potential and appropriate target for the prevention of wound progression following a burn.

The development of early burn-wound progression involves multiple mechanisms, including vasoconstriction/vasodilation, oxygen free radical-induced damage, hypoperfusion and microthrombosis, which activate inflammatory cascades and cell death (necrosis or apoptosis)^4^,^5^. Several previous studies have suggested that both free radical-induced oxidative stress and secondary mitochondria-related apoptosis play important roles in early burn-wound progression and prolonged inflammation^3^,^4^,^6^. Moreover, a series of signaling molecules, including phosphoinositide 3-kinase (PI3K), protein kinase B (Akt), and Bad (Bcl-xL/Bcl-2-associated death promoter homologue)^7^,^8^,^9^,^10^, modulate mitochondria-related apoptosis.

Astaxanthin (3,3′-dihydroxy-b,b’-carotene-4,4′-dione, ATX) is a natural carotenoid that is widely distributed in marine organisms such as algae, crustaceans, salmon, shrimp, and crabs, and it has more robust effects on quenching oxygen free radicals than other carotenoids^11^. According to a number of previous *in vitro* and *in vivo* studies, ATX protects against oxidative stress-induced cell or tissue damage^9^,^12^,^13^. In addition, the regulation of mitochondrial pathways is believed to be involved in the protective effect of ATX on burn-induced acute kidney injury, subarachnoid hemorrhaging, colon carcinogenesis, obesity, and cerebral or myocardial ischemia/reperfusion injury^8^,^9^,^14^,^15^,^16^. Given the crucial roles of oxidative stress and mitochondria-related apoptosis in early burn-wound progression, we hypothesized that ATX protects against burn-wound progression and explored the potential mechanisms of action of ATX.

## Results

### General and histological observation

**Figure 1:**
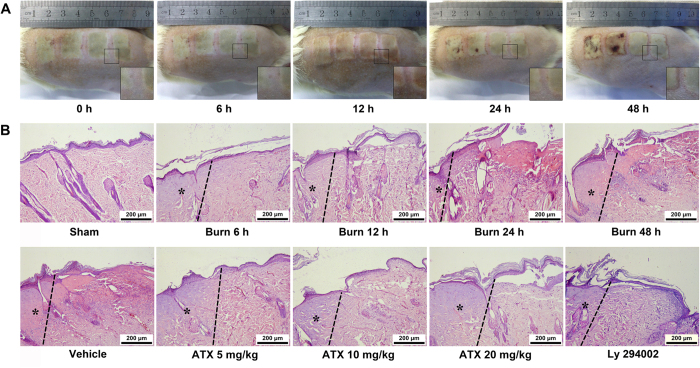
General observations and HE staining of burn models after burn injury or ATX administration. (**A**) Representative general images show progressive merging in the interspaces between two burn zones. (**B**) Representative HE-stained slices show the typical histological changes in the different groups. The progression boundary is marked with a dotted line, and the initial burn regions are labelled with asterisks, presenting obvious hyalinization. Scale bars = 200 μm, n = 8 per group. ATX, astaxanthin.

After burn injuries occur, the spaces between two burn zones tended to narrow and merge over time (Fig. 1A). Hematoxylin and eosin (HE) staining revealed some representative characteristics, such as a thinning of the epidermis, epithelial nuclei elongation, swelling of the dermis with collagen alteration, and inflammatory cell infiltration, and these effects increased over time after the burn. We also observed that the administration of ATX at all three doses alleviated the aforementioned changes. In contrast, the histological damage observed after Ly 294002 pretreatment was similar to that detected in the burn groups and differed from that found in the groups treated with ATX (Fig. 1B).

### ATX attenuated burn-induced oxidative stress in the zone of stasis of rat wounds

**Figure 2:**
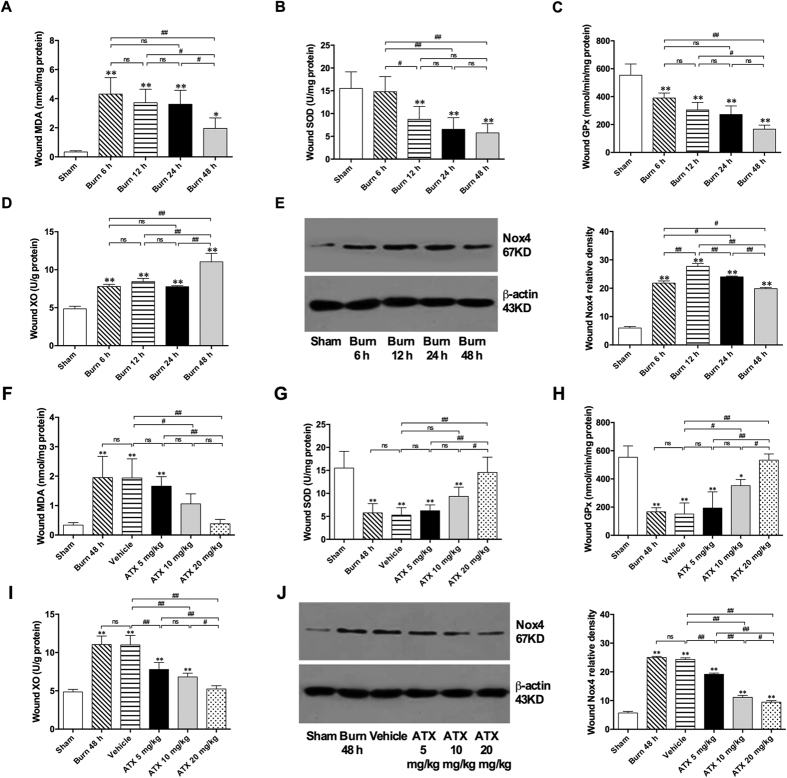
Assessment of oxidative stress within the zone of stasis and the effects of ATX. (**A**) Tissue MDA levels post burn. (**B**,**C**) Activities of SOD and GPx in tissues post burn. (**D**) Tissue levels of XO post burn. (**E**) Representative WB band and protein density of Nox4 from tissues post burn. (**F**) Tissue MDA levels after ATX administration. (**G**,**H**) Activities of SOD and GPx in tissues after ATX administration. (**I**) Tissue XO levels after ATX administration. (**J**) Representative WB band and protein density of Nox4 from tissues after ATX administration. The results are expressed as the means ± SDs. *p < 0.05 and **p < 0.01 versus the sham group; ^#^p < 0.05, ^##^p < 0.01, and ^ns^p > 0.05. n = 6 per group. ATX, astaxanthin; WB, western blot.

As an indicator of lipid peroxidation, the MDA level reflects the extent of oxidative damage. Within 6 h after burn injuries, MDA levels were significantly increased in the burn interspaces (Fig. 2A). Although this increase in the MDA levels was reduced 48 h post burn, it remained significant compared with levels in the sham group (Fig. 2A). An analysis of endogenous antioxidant enzymes revealed that the activity of superoxide dismutase (SOD) in wounds was markedly decreased 12, 24 and 48 h after the burn insults, but no significant difference was detected between two of the three time points (Fig. 2B). In addition, burn insults also induced a significant downregulation of glutathione peroxidase (GPx) levels at all four time points, with the lowest level detected 48 h after the burn (Fig. 2C). Both xanthine oxidase (XO) and the reduced form of nicotinamide adenine dinucleotide phosphate (NADPH) oxidase (Nox) contribute to the generation of reactive oxygen species. The ELISA results revealed that XO levels were markedly increased within 6 h post burn and remained steady until 24 h, whereas the 48-h post-burn group mostly presented significant elevations in XO levels (Fig. 2D). Moreover, Nox4, an important member of the Nox family, indicates mitochondrial status, and the present data demonstrate that Nox4 expression in the zone of stasis was significantly increased at all four selected time windows following burn injury and peaked at 12 h post burn (Fig. 2E).

Compared with the sham group, the vehicle group presented a significant increase in MDA levels, and this increase was similar to that found in the corresponding burn group (48 h; Fig. 2F). ATX treatment at a low dose (5 mg/kg) resulted in an insignificant decrease in MDA levels compared with those of the vehicle group; however, both the medium and high doses markedly decreased MDA levels (Fig. 2F). The burn-induced decreases in SOD and GPx activities were not influenced by the application of the vehicle solution (Fig. 2G and H). However, ATX exerted a dose-dependent effect, increasing SOD and GPx activities after burn injury, and peak activities were detected in the group treated with 20 mg/kg ATX (Fig. 2G and H). The increases in the expression of XO and Nox4 after a burn were markedly downregulated in correlation with increasing ATX doses (Fig. 2I and J).

### ATX ameliorated the inflammatory response in the zone of stasis

**Figure 3:**
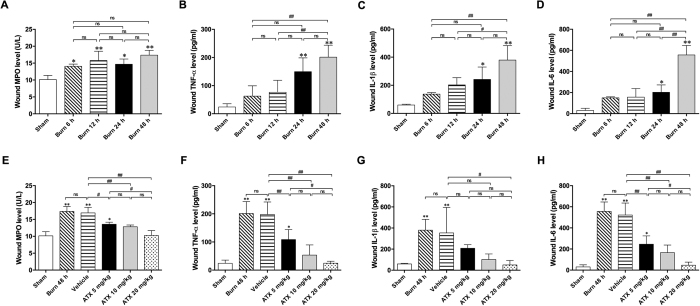
ATX attenuates the burn-induced increase in inflammatory mediator release in the zone of stasis. (**A**–**D**) Time-related changes in the tissue levels of MPO, IL-1β, IL-6 and TNF-α post burn. (**E**–**H**) Changes in the tissue levels of MPO, IL-1β, IL-6 and TNF-α after ATX administration. The results are expressed as the means ± SDs. *p < 0.05 and **p < 0.01 versus the sham group; ^#^p < 0.05, ^##^p < 0.01, and ^ns^p > 0.05. n = 6 per group. ATX, astaxanthin.

Commercial kits were used to test for the release of inflammatory mediators in the zone of stasis. We found significant elevations in the MPO levels at all four time windows after burn injuries, and the 12- and 48-h groups presented more marked changes (Fig. 3A). The analysis of inflammatory cytokines revealed a time-dependent increase in TNF-α, IL-1β or IL-6 levels in burn wounds, with significant changes detected 24 h and a peak observed at 48 h post burn (Fig. 3B–D).

We then verified the effect of ATX on inflammation in burn wounds. The present results indicate that vehicle treatment did not change MPO levels in the zone of stasis compared with levels in the 48 h post-burn group (Fig. 3E). All three ATX doses reduced the elevated MPO levels observed after burn injury, and the 20 mg/kg dose exerted the most significant effect (Fig. 3E). Additionally, similar levels of TNF-α, IL-1β and IL-6 were found in the 48-h post-burn and vehicle groups. In contrast, we observed that ATX treatment attenuated the burn-induced increases in TNF-α or IL-6 levels in a dose-dependent manner, whereas only high doses of ATX markedly decreased elevated IL-1β levels in the zone of stasis (Fig. 3F–H).

### ATX dose-dependently reduced cell apoptosis in the burn wound tissue of rats

**Figure 4:**
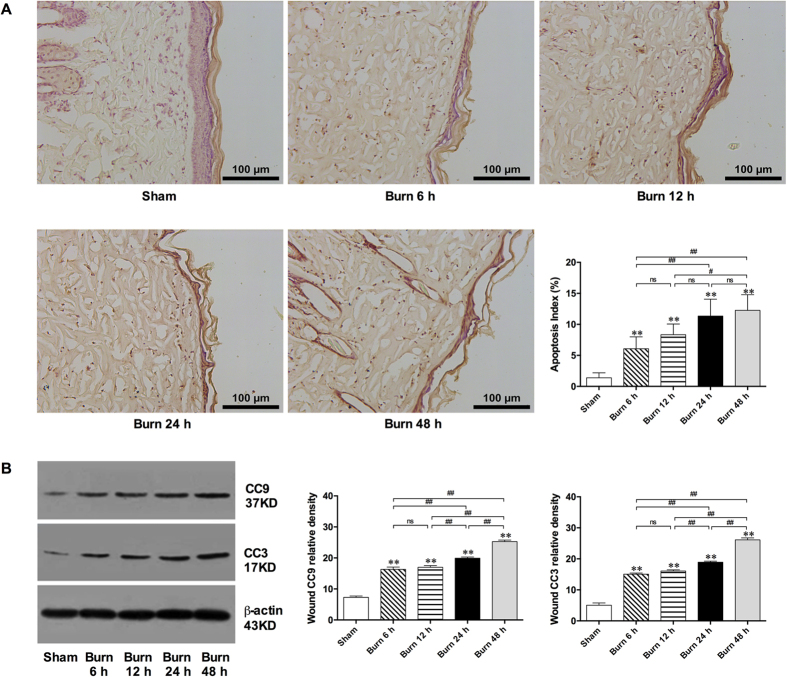
Apoptosis in the zone of stasis over time. (**A**) Representative images of TUNEL-stained slices (brown-labelled nuclei showed the apoptotic cells) and apoptotic cells counted after burn injury. (**B**) WB results for CC9 and CC3 post burn. Scale bars = 100 μm. The results are expressed as the means ± SDs. *p < 0.05 and **p < 0.01 versus the sham group; ^#^p < 0.05, ^##^p < 0.01, and ^ns^p > 0.05. n = 6 per group. ATX, astaxanthin; WB, western blot; CC3, cleaved caspase 3; CC9, cleaved caspase 9.

**Figure 5:**
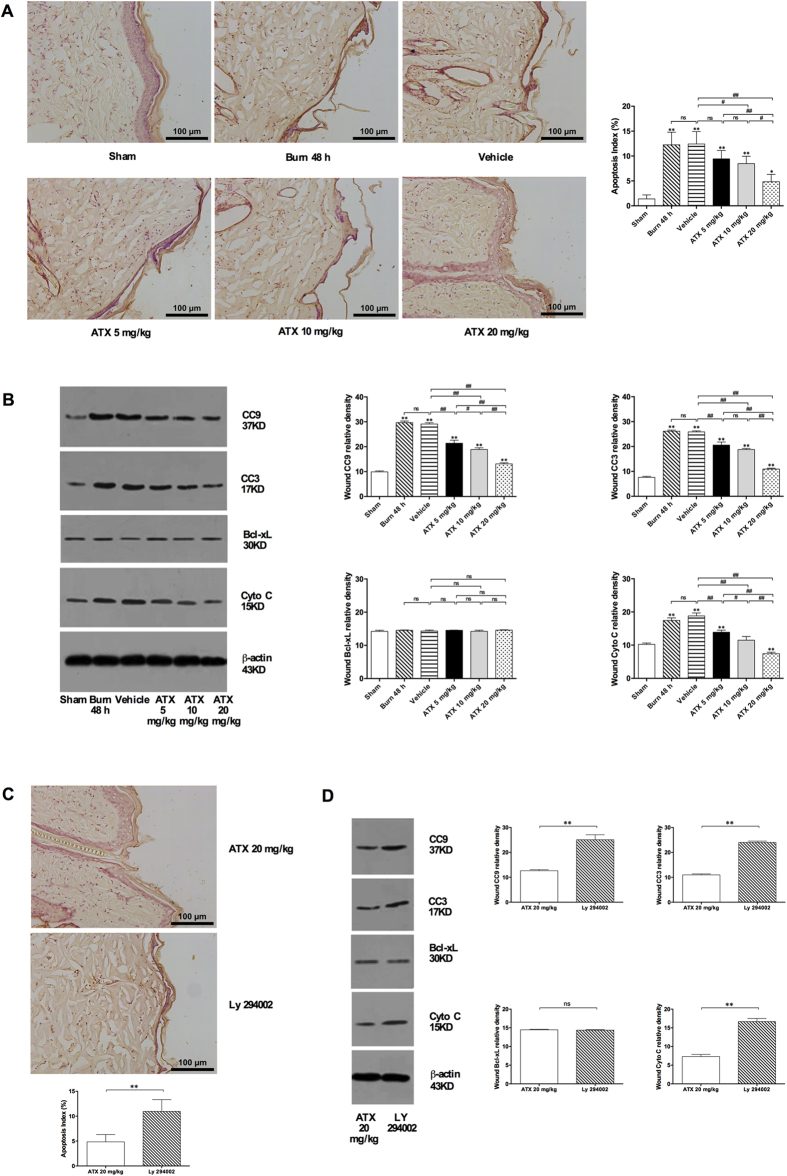
ATX ameliorates apoptosis in the zone of stasis and the upregulated expression of proapoptotic proteins post burn, and these effects can be reversed by Ly 294002. (**A**) Representative images of TUNEL-stained slices and the ratio of apoptotic cells after ATX treatment. (**B**) WB results for CC9 and CC3 after ATX treatment. (**C**) Representative images of TUNEL staining in the groups treated with 20 m/kg ATX and Ly 294002. (**D**) WB results for CC3, CC9, Bcl-xL and Cyto C in the groups treated with 20 m/kg ATX and Ly 294002. Scale bars = 100 μm. The results are expressed as the means ± SDs. *p < 0.05 and **p < 0.01 versus the sham group; ^#^p < 0.05, ^##^p < 0.01, and ^ns^p > 0.05. n = 6 per group. ATX, astaxanthin; WB, western blot; CC3, cleaved caspase 3; CC9, cleaved caspase 9; Cyto C, cytochrome C.

According to observations and assessments of TdT-mediated dUTP nick end labeling (TUNEL)-stained slices, the number of apoptotic cells with brown-labeled nuclei in burn wounds increased over time (Fig. 4A). Moreover, the protein expression of cleaved caspase 3 (CC3) and 9 (CC9), which are important signaling molecules in the mitochondria-apoptotic pathway, increased gradually from 6 to 48 h post burn in the interspace skin (Fig. 4B). The administration of ATX resulted in a dose-dependent reduction in the number of apoptotic cells (Fig. 5A) compared with the number observed in the vehicle group. Moreover, all three ATX doses significantly decreased CC3 and CC9 expression levels, and the highest dose (20 mg/kg) exerted the strongest effect (Fig. 5B). To assess the upstream signals in the mitochondria-related apoptotic pathway, B-cell lymphoma (Bcl)-xL and cytochrome C (Cyto C) were selected for Western blot analysis. At 48 h after a burn insult, an insignificant change in Bcl-xL expression and an obvious increase in Cyto C expression were detected (Fig. 5B). Even higher doses of ATX did not effect Bcl-xL expression (Fig. 5B). However, the increased expression of Cyto C post burn was dose-dependently reduced by ATX administration, whereas no statistically significant difference was observed between the 48-h post-burn and vehicle groups (Fig. 5B).

### Ly 294002 reversed the ATX-mediated relief of apoptosis in burn wounds

Ly 294002, a specific PI3K inhibitor, was used to identify possible mechanisms of apoptosis in the zone of stasis during burn-wound progression. We hypothesized that wound progression might involve the PI3K/Akt/Bad/caspase signaling cascade, a typical mitochondria-related apoptotic pathway. TUNEL staining indicated that Ly 294002 pretreatment clearly increased the number of positively labeled (brown nuclei) apoptotic cells in the tested zone after treatment with 20 mg/kg ATX (Fig. 5C). Moreover, Ly 294002 also reversed the reduction in mitochondria-related pro-apoptotic proteins (CC 3/9 and Cyto C) in the burn wound caused by a high-dose (20 mg/kg) of ATX (Fig. 5B). However, Ly 294002 did not influence Bcl-xL expression levels (Fig. 5D).

### ATX upregulated the phosphorylation of Akt and Bad in the burn interspace tissues of rats

**Figure 6:**
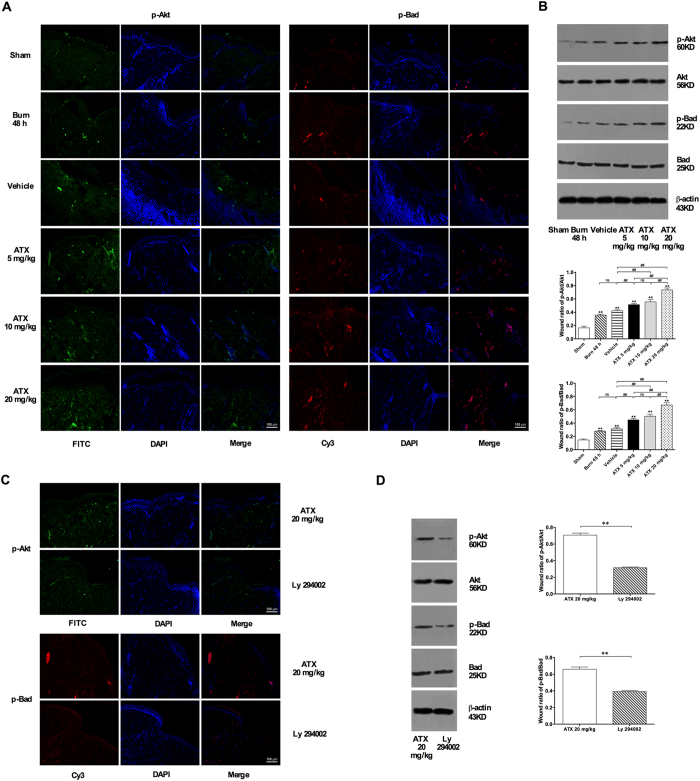
ATX dose-dependently increases the phosphorylation of Akt and Bad in burn wounds, and Ly 294002 reverses the increased distribution and expression of p-Akt and p-Bad after high-dose ATX administration. (**A**) Representative images of immunofluorescence staining of p-Akt and p-Bad after ATX application. (**B**) WB bands and relative p-Akt/Akt and p-Bad/Bad ratios after ATX treatment. (**C**) Representative images of immunofluorescence staining of p-Akt and p-Bad in groups subjected or not subjected to Ly 294002 pretreatment. (**D**) WB bands and relative p-Akt/Akt and p-Bad/Bad ratios in the groups subjected or not subjected to Ly 294002 pretreatment. Scale bars = 100 μm. The results are expressed as the means ± SDs. *p < 0.05 and **p < 0.01 versus the sham group; ^#^p < 0.05, ^##^p < 0.01, and ^ns^p > 0.05. n = 6 per group. ATX, astaxanthin; WB, western blot.

Immunofluorescence (IF) staining revealed that the distributions of phosphorylated Akt (p-Akt, FITC, green-labeled)- and Bad (p-Bad, Cy3, red-labeled)-positive skin cells were increased post burn in both the burn (48 h) and vehicle groups (Fig. 6A). ATX administration further enhanced p-Akt and p-Bad immunoreactivity, and the highest numbers of p-Akt- and p-Bad-positive cells were observed in the group treated with 20 mg/kg ATX (Fig. 6A). The Western blot results demonstrated increased p-Akt and p-Bad expression levels in both the burn (48 h) and vehicle groups (Fig. 6B). Moreover, increasing doses of ATX resulted in the further upregulation of p-Akt and p-Bad expression, with a peak observed in the group treated with a dose of 20 mg/kg (Fig. 6B).

### Ly 294002 reversed the ATX-mediated upregulation of Akt and Bad phosphorylation in the cutaneous zone of stasis post burn

The increased distributions of green-labeled (FITC) p-Akt and red-labeled (Cy3) p-Bad associated with ATX (20 mg/kg) treatment in burn wounds were decreased by Ly 294002 pretreatment (Fig. 6C). Furthermore, the Western blot analysis results demonstrated that Ly 294002 significantly reversed the ATX-induced (20 mg/kg) elevation of Akt and Bad phosphorylation in the zone of stasis (Fig. 6D).

## Discussion

**Figure 7:**
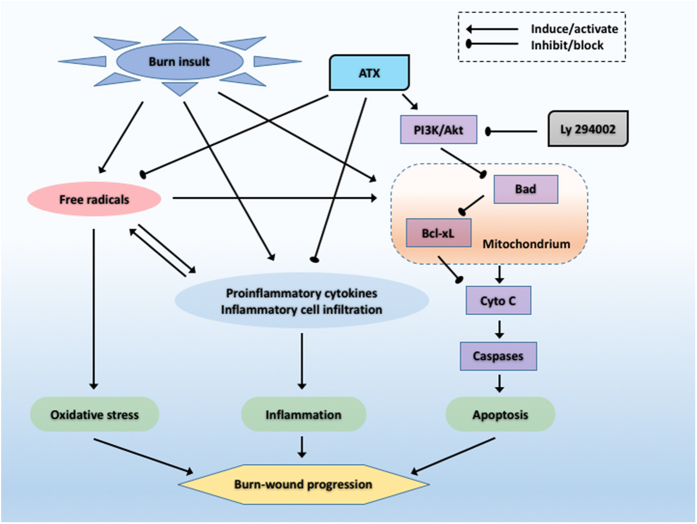
Schematic diagram of the potential effects of ATX. ATX, astaxanthin; Cyto C, cytochrome C.

After burn insults, multiple factors, such as ischemia, oxidative stress, inflammation, and cell death (necrosis or apoptosis), contribute to the conversion of early burn wounds, which is a progressive change in the peri-burn zones that includes stasis and hyperemia. Conversion might lead to a deepening or extension of the initiating location^3^,^4^,^6^,^17^. In view of the series of disastrous results following burn-wound progression, such as hypertrophic scarring, wound contractures, infections, and sepsis, the regimen and occasion for appropriate intervention have received increased interest from researchers^18^. Considering its reported effects on oxidative stress, apoptosis and inflammation, ATX might be of value during the transformation of the zone of stasis^8^,^12^,^14^,^19^. In the present study, we first investigated the potential effect of ATX on burn wound progression. The results indicate the following: 1) ATX alleviates burn-induced histological changes in the burn wound; 2) ATX dose-dependently attenuates oxidative stress during the early stages following a burn in response to the production of free radicals by inhibiting lipid peroxidation and activation of the NADPH-dependent oxidase system and by enhancing the activity of endogenous antioxidant enzymes; 3) ATX could relieve inflammation during the early stages in burn wounds; and 4) increasing doses of ATX further reduce cell apoptosis in the zone of stasis by influencing the mitochondria-related apoptotic pathway. A summary of the effects of ATX is shown in Fig. 7.

First, in addition to the general changes, we observed a series of histological changes in progressive burn wounds, such as thinning of the epidermis, epithelial nuclear elongation, swelling of the dermis with collagen alterations, and inflammatory cell infiltration. In this study, the administration of varied doses of ATX appeared to attenuate these changes to different degrees, as demonstrated through microscopic observations of HE-stained slices. These findings suggest a protective effect of ATX on burn-wound progression. To confirm the detailed mechanisms underlying this effect, we further explored the potential effects of ATX on oxidative stress, inflammation and apoptosis.

Free radicals, which are molecules with unpaired outer-orbit electrons, contribute to many physiological processes based on their powerful oxidizing or reducing capacities^1^,^20^. In general, reactive oxygen species (ROS) include several types of free radicals, such as superoxide radicals (O_2_−), hydrogen peroxide (H_2_O_2_), and hydroxyl radicals (∙OH), and a balance between ROS and the antioxidant system is observed under normal conditions^21^. When this balance is disrupted by trauma or disease, oxidative stress causes tissue injury. Accompanied by the overproduction of free radicals, multiple factors contribute to burn-induced oxidative stress. On the one hand, as burn injuries progress, the thermal energy generated can directly generate free radicals via homolytic bond fission^22^, and on the other hand, the activation of polymorphonuclear neutrophil leukocytes (PMNLs), xanthine oxidase (XO) and NADPH oxidase (Nox) is also involved in the production of free radicals^23^. Oxidative stress has been demonstrated to contribute to local inflammation and tissue cell apoptosis in burn wounds and other organs^4^. Mitochondria are cellular organelles that are sensitive to ROS, and the function of mitochondrial membranes can be damaged by ROS-induced lipid peroxidation, resulting in the release of Cyto C^6^. Furthermore, inflammatory cytokines, such as TNF-α, can also enhance the activity of Nox, leading to the production of ROS^24^. In addition, ROS can largely consume endogenous antioxidant enzymes (such as SOD and GPx), which might reduce the functionality of the inner antioxidant defense system^1^. Prior studies on burn wounds have found that ROS participate in progressive tissue destruction in the zone of stasis^4^,^25^. Therefore, ROS-induced oxidative stress is a potential therapeutic target for preventing burn wound progression. As a natural and powerful antioxidant, ATX exerts protective effects against tissue and organ injuries by reducing oxidative stress and affecting the activities of antioxidant enzymes or oxidases (XO or Nox) that otherwise contribute to the production of free radicals^8^,^14^. Our previous study, which investigated the effect of ATX on acute kidney injury in burned rats, revealed that ATX reduces MDA levels and increases the activities of inner antioxidant enzymes in renal tissue after burns in a dose-dependent manner^8^. Other studies have demonstrated that the administration of ATX has a similar effect on other organ injuries after severe traumatic injury^8^,^9^,^26^. Moreover, using a rat model of live ischemia-reperfusion injury (I/R), GD Curek *et al*. observed that ATX treatment attenuated I/R-induced elevations in circulating XO by decreasing the hepatic conversion of xanthine dehydrogenase (XDH) to XO^27^. For Nox, Q Ye *et al*. reported that ATX protects against MPP(+)-induced oxidative stress in PC cells and that this effect is mediated via the regulation of Nox with the assistance of heme oxygenase-1 (HO-1)^28^. In the current study, we observed that medium and high doses of ATX appear to be effective in ameliorating lipid peroxidation and improving the activities of SOD and GPx. The effect was dose-dependent and peaked at 100 mg/kg. Furthermore, the effect of ATX might be mediated by XO and Nox. In this study, we found that the XO and Nox4 levels in post-burn wounds increased over time. Even a small dose of ATX decreased XO and Nox4 levels, and higher doses further enhanced this downregulation. These data show that ATX might protect against burn-wound progression by attenuating ROS-induced oxidative stress, an effect that might also involve the regulation of free radical production by influencing XO and Nox.

Local and systemic inflammation, which are physiological responses to external stress, are usually stimulated after burn injuries and contribute to cutaneous or organ injuries caused by thermal insults. In burn wounds, a prolonged inflammatory response, which is mediated by complement system activation, inflammatory mediator release, delayed inflammatory cell apoptosis, inflammatory signals and ROS, participates in burn-wound progression^4^,^6^. Polymorphonuclear leukocytes (PMNLs) are the first leukocytes to arrive at the location of a burn wound in the early stages following burn insults, and these cells exacerbate tissue injuries, prolong wound healing, and increase the systemic inflammatory response after a severe burn injury^1^. In this study, we selected MPO levels as an index to reflect the infiltration of PMNLs and observed a significant time-dependent elevation in burn wounds, which is consistent with prior results. In addition, PMNLs and subsequently activated immunocytes, such as macrophages, can secrete pro-inflammatory mediators, including TNF-α, IL-1β and IL-6, which are involved in the development of local or systemic inflammation^1^,^25^. In agreement with previous studies, we further found consistent increases in the levels of pro-inflammatory cytokines (TNF-α, IL-1β and IL-6) in the regions adjacent to burn wounds over time^25^. Additionally, the reported reductions in factors affecting wound progression, which primarily involve inflammation secondary to a burn, also demonstrate that decreasing the release of pro-inflammatory mediators can effectively attenuate burn-wound conversion by inhibiting the adhesion and infiltration of inflammatory cells^25^,^29^,^30^,^31^,^32^. With respect to ATX, J Cao *et al*. suggested that a diet supplemented with ATX could decrease the elevated levels of MPO induced by aflatoxin-B1 in liver tissue of broliers^33^. Moreover, a series of *in vivo* and *in vitro* studies revealed that ATX administration decreases the release of TNF-α, IL-1β or IL-6^13^,^16^,^19^,^34^. Based on results from human blood testing, JS Park *et al*. posited that daily supplementation with ATX could downregulate the level of inflammatory cytokines, such as IL-6 and IFN-gamma, and inhibit reactive oxygen and nitrogen species^35^. Our present study also shows that ATX dose-dependently reduces tissue TNF-α, IL-1β and IL-6 levels in the zone of stasis. In summary, ATX displays an anti-inflammatory effect that is mediated by decreasing PMNL infiltration and cytokine release, and this effect might be a possible mechanism underlying the protective effect of ATX against burn-wound conversion.

Cell death also plays a crucial role in the early development of burn wounds, and three types of cell death, namely, necrosis, apoptosis, and autophagy, exist^1^,^4^. Previous studies have suggested that both necrosis and apoptosis are involved in the pathogenesis of burn-wound conversion, whereas the specific role of autophagy remains controversial^25^,^36^,^37^. Therefore, we focused on investigating the possible effect of ATX on factors influencing necrosis and apoptosis. Necrosis is primary a pathological change in the zone of coagulation, which undergoes a direct thermal insult^3^,^6^. Some researchers have suggested that instead of the pathological description “necrosis”, the term “oncosis” should be used to specify the process of cell death accompanied by cellular and organelle swelling, membrane permeability, and the depletion of energy stores^6^. In the zone of stasis, oncosis can be secondary to the overproduction of ROS, inflammation, and weak circulation, which eventually exacerbates the condition of the peri-burn region^6^. Considering the generally irreversible characteristics of oncosis, preventing this process primarily involves reducing the factors that initiate oncosis^5^. The aforementioned data show that ATX might prevent oncosis in the dermis by attenuating inflammation and oxidative stress. In contrast to oncosis, apoptosis, which is defined as programmed cell death, plays a more important role in the initial post-burn stage and contributes to cell death in the zone of stasis^5^,^6^. Our and previous studies have revealed that ongoing apoptosis in the zone of stasis contributes to tissue loss and structural deterioration, and the treatment of this zone would be beneficial for burn-wound healing^25^. With the exception of TUNEL staining, which directly shows apoptotic cells, caspases are one of the most sensitive markers of apoptosis. Caspases can be categorized as initiators (typically caspase 9) or effectors (typically caspase 3), and the activation of initiator caspases leads to the subsequent cleavage of internal aspartic acid residues of effector caspases and finally to their activation^6^,^38^. The activated caspase cascade causes morphological and biochemical changes that are characteristic of apoptosis in biological tissues through the proteolytic cleavage of target proteins^38^. Our study showed a significant elevation in the number of apoptotic cells in burn wounds over time, and this effect parallels the marked upregulation of CC9 or CC3 expression. Furthermore, the decreases in apoptotic cell numbers and CC9 or CC3 expression revealed that ATX treatment has a dose-dependent effect on relieving apoptosis in the zone of stasis. Moreover, ATX significantly downregulated the increased expression of Cyto C in burn wounds, indicating that mitochondria might be involved in regulating the role of ATX in apoptosis. Cyto C is commonly released by mitochondria under cellular stress caused by insults and can combine with Apaf-1 and caspase 9 to form an activated complex, which finally cleaves caspase 3 into CC3 (an activated state) and induces apoptosis^38^. As a member of the antiapoptotic Bcl-2 subfamily, Bcl-xL usually combines with Bad to build a proapoptotic complex, which increases the permeability of the mitochondrial membrane by acting on voltage-dependent anion channels, resulting in the release of Cyto C from mitochondria^39^. In the current study, we did not find a marked change in Bcl-xL expression after ATX treatment, which might indicate that the effect of ATX is mediated by the proapoptotic Bcl-2 subfamily rather than the antiapoptotic subfamily. Furthermore, according to several studies based on rodent models, ATX administration effectively attenuates cell apoptosis secondary to brain jury or burn-induced kidney injury by regulating the Akt/Bad/Cyto C signaling cascade^8^,^9^. Therefore, we investigated the possible roles of Akt and Bad in burn-wound progression. The results of immunofluorescence staining and Western blot assays revealed that Akt and Bad phosphorylation significantly increased after burn insult, which results from an inner defense mechanism. Additionally, ATX could further induce Akt and Bad phosphorylation. Phosphorylated Akt is commonly in an activated status, which can prompt Bad to dissociate from Bcl-xL through phosphorylation of the Serine-136 residue of Bad^39^,^40^. All of the above-mentioned results suggest that the mitochondria-apoptotic signaling pathway mediates the beneficial effect of ATX on cell apoptosis during burn-wound progression. To further verify the role of Akt/Bad/Cyto C/caspases, we treated rats with Ly 294002, a PI3K inhibitor, to determine the possible mechanism of ATX on burn-wound conversion. The present study revealed that Ly 294002 pretreatment could abolish the effect of high-dose ATX treatment on cell apoptosis in the zone of stasis and influence the Akt/Bad/Cyto C/caspase signaling cascade, supporting our proposed role of ATX-mediated protection against burn-wound progression via mitochondria-related apoptosis.

Multiple forms of ATX products, such as a capsule, soft gel, tablet, powder, biomass, cream, energy drink, oil or extract, are available on the market^41^. The application of these products focuses on the prevention of bacterial infection, inflammation, vascular failure, cancer, and cardiovascular diseases, the inhibition of lipid peroxidation, the amelioration of cell damage and the improvement of brain/liver function and skin thickness^14^,^34^,^41^,^42^,^43^,^44^,^45^. For burn patients, ATX should be administered via different routes based on the degree of injury or the patients digestive function. Patients with a limited burn area who may still have a functional digestive system could be treated with ATX alone or in combination with enteral nutrition via the oral route or a nasogastric tube. Although previous clinical trials investigating different populations or patients have recommended an oral ATX dose ranging from 2 mg/day to 100 mg/day, the dose-related effect of ATX on burn patients should be explored through randomized clinical research^41^. In contrast, burn patients with severe burn injuries may require other routes of ATX administration, such as the local or intravenous treatments, based on their degree of digestive dysfunction and the speed of drug distribution in burn wounds; further investigations could focus on the development of proper solvents and on local ATX penetration. Previous studies have demonstrated that burn-wound progression could start in the early stages after a burn; therefore, the timing of ATX administration might need to be adjusted to in relation to the specific condition^1^,^4^,^25^. In cooperation with a clinical pharmacy, a pharmacodynamics-based study could provide strong support to clinical trials of ATX application.

In conclusion, the present study demonstrated that ATX, a natural antioxidant, exerts a protective effect against burn-wound conversion by attenuating free radical-related oxidative stress and subsequent inflammation or cell death. The mitochondria-related apoptotic pathway is involved in regulating the role of ATX in apoptosis within the zone of stasis. Based on the results from this and our prior study, ATX has the potential to be a novel, safe therapy that prevents burn wounds from deepening and extending their range during the early stages following injury.

## Methods

### Experimental animals and “comb” burn model

The present study was performed according to protocols approved by the Committee on Animal Care and Use of the Second Affiliated Hospital, School of Medicine, Zhejiang University (2016–144), and strictly followed the National Institutes of Health Guidelines for the Care and Use of Laboratory Animals. Adult male Sprague-Dawley rats (weighing approximately 250–300 g) were purchased from the Shanghai Slac Laboratory Animal Company (Shanghai, China). These animals were housed in a filtered-air unit with a 12-h light/dark cycle and constant temperature and humidity and were given free access to food and water.

The “comb” burn model was established in accordance with previous reports^25^,^46^. Specifically, a custom-made rectangular brass comb (with a transverse section of approximately 20 mm × 10 mm) was boiled in 100 °C water for 5 min and then applied to the shaved skin surface of the rat’s dorsum for 20 s after anesthesia was induced (sodium pentobarbital, Sigma, St. Louis, MO, USA, 50 mg/kg, intraperitoneal). A row of four bands of full-thickness burns (20 mm × 10 mm) was made with three interspaces of uninjured skin (20 mm × 5 mm) to represent the zone of stasis. The burn wound area occupied approximately 4% of the total body surface area (TBSA). For the sham group, the brass comb was heated in 25 °C water and applied to the shaved rat dorsum after anesthesia. During the operation, the breathing and heart rates of the burned rats were carefully monitored to ensure that all of the rats were under anesthesia and pain-free before being allowed to recover from the anesthesia. Moreover, all rat models were housed in individual cages and given 0.25 mg/kg buprenorphine via subcutaneous injections immediately and every 12 h after the burn injury for analgesia. Previously reported pain and distress scales were used in the evaluation of pain in the burned rats immediately and every 6 h after recovering from anesthesia; these scales informed pain-relieving therapy^8^.

### Animal grouping and research design

**Figure 8:**
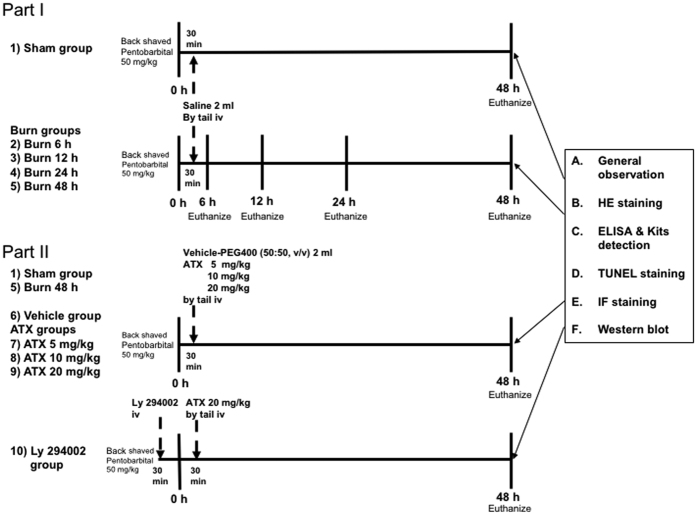
Experimental design and animal grouping.

Eighty animals were randomly assigned to ten groups: the sham group (n = 8), four burn groups (n = 8 per group), three ATX-treatment groups (n = 8 per group) and the burn plus vehicle (n = 8) and Ly 294002 (n = 8) groups. The animals in the three ATX-treatment groups received ATX (Sigma-Aldrich, St. Louis, MO, USA) dissolved in polyethylene glycol 400-N,N-dimethylacetamide (PEG400), purchased from Sigma-Aldrich (St. Louis, MO, USA; 50:50, v/v), at dosages of 5 mg/kg, 10 mg/kg and 20 mg/kg (all in 2 ml) via tail intravenous injection (iv). The rats in the sham and four burn groups were given equal volumes (2 ml) of 0.9% saline via tail iv. The rats in the vehicle group were given equal amounts of PEG400 (50:50, v/v, 2 ml) without any drugs via tail iv. All of the above-mentioned treatments were administered 30 min after surgery, and both the dosages and the method of ATX administration were selected according to previous studies^8^,^11^. In addition to 20 mg/kg of ATX, the rats in the Ly 294002 group were administered Ly 29400 (Sigma-Aldrich, St. Louis, MO, USA; dissolved in phosphate-buffered saline (PBS) with 1% DMSO) at a dose of 0.3 mg/kg via tail iv^8^,^47^. The four burn groups were euthanized using an overdose of sodium pentobarbital at 6, 12, 24 or 48 h post burn, whereas the other groups were euthanized at 48 h post burn or after the sham operation. One band of the interspace skin with 2 mm of burned tissue on each side (20 mm × 9 mm) was harvested from each burned rat and stored in 4% paraformaldehyde at 4 °C for subsequent histological and immunofluorescence analyses, and the remaining two bands of interspace skin (without burn tissue; 20 mm × 5 mm) from each burned rat were stored at −80 °C for analysis using commercial kits and Western blot assays. The corresponding bands of unburned skin from each rat in the sham group were also collected as control samples. The general design of this study is shown in Fig. 8.

### Histological preparation

Skin samples were fixed in 4% paraformaldehyde, embedded in paraffin and sectioned at a thickness of 3 μm using a rotary microtome (RM2245, Leica, Solms, Germany). All slices were deparaffinized and rehydrated prior to subsequent staining.

### Histological examination

Hematoxylin and eosin (HE) staining was performed for histological examinations, and the tissue slices were observed and recorded under a microscope (DM2500, Leica, Solms, Germany). Five visual fields (×200) from every slice were randomly selected for analysis.

### TUNEL staining

TUNEL staining was conducted with a commercial cell-death detection kit purchased from Roche Diagnostics (Indianapolis, IN, USA) according to the manufacturer’s recommended protocol. The stained slices were observed under a microscope (DM2500, Leica, Solms, Germany), and images were recorded. The index of apoptosis was calculated as the percentage of apoptotic cells among all the cells counted under blinded conditions. Two independent investigators, who were blinded to the group assignments, were involved in calculating the index. At least three visual fields per slide and five slides per group were evaluated by the investigators.

### Immunofluorescence staining

Prepared slices were washed in PBS for 10 min and then boiled in a 0.01 mmol citrate buffer (pH = 6) for 10 min for antigen retrieval. After incubation with hydrogen peroxide for 10 min, 5% bovine serum albumin (BSA) was applied as the blocking solution for 20 min at room temperature. Without being washed, the sections were incubated with anti-p-Akt (1:200; #4060, Cell Signaling Technology, MA, USA) or anti-p-Bad (1:200; sc-12969-R, Santa Cruz, CA, USA) antibodies overnight at 4 °C. After being rinsed with PBS, the sections were incubated with FITC (1:50; BA1105, Boster, Wuhan, China)- or Cy3 (1:50; BA1032, Boster, Wuhan, China)-labeled goat anti-rabbit secondary antibodies for 2 h at 37 °C in the dark. The sections were rinsed and stained with DAPI (100 ng/ml; Boster, Wuhan, China) for 8 min at room temperature and then mounted with Vectashield mounting medium (Boster, Wuhan, China). All slices were observed and photographed under a fluorescence microscope (DM5500B, Leica, Solms, Germany).

### Oxidative stress assessment

Skin tissue homogenates from the burn wounds were reacted with a thiobarbituric acid reactive species (TBARS) assay kit (KGT003–1, KeyGEN, Nanjing, China) to determine the MDA levels, which are expressed as nmol/mg protein. Tissue superoxide dismutase (SOD) activities in the skin from burn wounds were evaluated and measured using commercial assay kits from KeyGEN Biotech (KGT00150, Nanjing, China) according to the manufacturer’s recommended protocol. The results are expressed as U/mg protein. The absorbance values were measured using a microplate reader (Model 680 Microplate Reader, BIO-RAD, CA, USA).

### ELISA assessment

Skin tissue homogenates were obtained using a glass homogenizer and were used for the detection of inflammatory mediators (MPO, IL-1β, IL-6, and TNF-α) via ELISA. The levels of these mediators were measured using commercial assay kits purchased from KeyGEN Biotech (IL-1β: KGERC007, IL-6: KGERC003, TNF-α: KGERC102a, Nanjing, China) and Lianshuo Biological (MPO: AE90769Ra, Shanghai, China).

### Western blot analysis

Frozen skin samples were cut into pieces and lysed with RIPA lysis buffer (AR0105, Boster, Wuhan, China) for 1 h on ice. The lysates were mixed with loading buffer and centrifuged at 14,000 *g* for 10 min, and the protein samples were subjected to SDS-PAGE and then transferred onto nitrocellulose membranes via electrophoresis. In addition, aliquots of the samples were used to determine the protein concentration of each sample using a bicinchoninic acid (BCA) kit (KGPBCA, KeyGEN, Nanjing, China). Subsequently, the membranes were incubated in blocking buffer for 2 h and incubated overnight at 4 °C with the following primary antibodies: anti-NOX4 (1:2000; ab109225, Abcam, Cambridge, UK), anti-p-Akt (1:500; sc-33437, Santa Cruz, CA, USA), anti-Akt (1:500; sc-8312, Santa Cruz, CA, USA), anti-p-Bad (1:1000; sc-12969-R, Santa Cruz, CA, USA), anti-Bad (1:1000; sc-8044, Santa Cruz, CA, USA), anti-cleaved caspase 9 (1:1000; #9509, Cell Signaling Technology, MA, USA), anti-cleaved caspase 3 (1:1000; #9664, Cell Signaling Technology, MA, USA), anti-Bcl-xL (1:800; sc-7195, Santa Cruz, CA, USA), and anti-cytochrome C (1:1000; sc-7159, Santa Cruz, CA, USA). β-actin (1:2000; SC-47778, Santa Cruz, CA, USA) was used as a control on the same membranes. After the application of secondary antibodies, the bands were detected with West Dura Extended Duration Substrate (Pierce, USA), and exposed X-ray films (Kodak, USA) were analyzed using Bandscan 5.0 software based on comparisons with β-actin bands.

### Statistical analysis

The data are presented as the means ± standard deviations (SDs). GraphPad Prism version 5.02 (San Diego, CA, USA) was used for the statistical analyses. Multiple comparisons were performed via one-way analysis of variance (ANOVA) followed by Bonferroni post hoc tests. Unpaired t-tests were used for comparisons between two groups. P-values less than 0.05 were considered statistically significant.

## Additional Information

**How to cite this article**: Fang, Q. *et al*. Astaxanthin protects against early burn-wound progression in rats by attenuating oxidative stress-induced inflammation and mitochondria-related apoptosis. *Sci. Rep.* **7**, 41440; doi: 10.1038/srep41440 (2017).

**Publisher's note:** Springer Nature remains neutral with regard to jurisdictional claims in published maps and institutional affiliations.
